# Implementing digital-supported team-based learning for large undergraduate cohort in a resource-limited setting: a pilot study developed through an international academic partnership

**DOI:** 10.1186/s12909-026-09409-y

**Published:** 2026-05-09

**Authors:** Neranja Fonseka, Chathura Ratnayake, Vasanthi Pinto, Kosala Marambe

**Affiliations:** Faculty of Medicine, University of Peradeniya, Kandy, Sri Lanka

**Keywords:** Team-based learning, Pear Deck, Medical education, International academic partnership, Large group teaching, Active learning, Educational technology, Resource-limited settings

## Abstract

**Background:**

Growing student numbers and limited teaching resources, especially in low- and middle-income countries (LMICs) such as Sri Lanka, hinder the delivery of interactive, student-centred medical education. Traditional lectures alone do not adequately support clinical reasoning, pointing to the need for active, application-focused methods aligned with technology-shaped learning expectations. Team-based learning (TBL) encourages collaboration and accountability, but can be resource-intensive and is not well-documented in large cohorts in LMIC settings. This study investigates a technology-supported TBL session delivered by a single lecturer, focusing on student perceptions and the mentoring within an international academic partnership.

**Method:**

After a faculty development programme with the University of Illinois Chicago introduced staff to TBL principles, a single-session pilot TBL intervention was implemented at the Faculty of Medicine, University of Peradeniya, as a descriptive service evaluation at Kirkpatrick Level 1. An existing lecture for third-year medical students was redesigned as a TBL session using the available infrastructure. In line with TBL principles, 252 students were assigned to 20 balanced teams. Eight abnormal uterine bleeding case scenarios and preparatory materials were provided two weeks in advance. During the session, students completed individual and group readiness assurance tests using Pear Deck for real-time interaction. One lecturer facilitated the discussion and completed it within two hours. Student perceptions were collected using a ten-item Likert-scale questionnaire and free-text comments.

**Results:**

Among 252 students, 209 participated (82.9%), and 191 of them completed the survey (91.4%). The median Likert-scale score was 4 or higher for engagement, teamwork, and use of the digital platform. Preparation-related items were scored lower. Free-text responses described the session as interactive, engaging, and helpful for maintaining attention and encouraging reflection.

**Discussion:**

This pilot study indicates that a technology-supported TBL session is feasible for a large cohort in a resource-limited setting, with strong reported engagement. Students responded positively, despite varying levels of preparation, and the digital platform was a feasible and acceptable tool for teamwork. Limitations included large team sizes, fixed seating, and the inability to use certain standard TBL tools. Further refinement, faculty development, and curricular integration are considered.

**Supplementary Information:**

The online version contains supplementary material available at 10.1186/s12909-026-09409-y.

## Background

The growing number of students entering medical schools has made it increasingly difficult to deliver teaching that is truly interactive and centred on learners’ needs. These concerns are particularly evident in low- and middle-income countries (LMICs), where increasing enrolment often outpaces available resources, such as teaching spaces, educational technologies, and trained staff. Moreover, the availability of knowledge on mobile devices has changed students’ expectations about how they should learn. Taken together, these developments highlight the need to move beyond traditional teaching practices and explore approaches that actively encourage students to engage more proactively in their learning.

Sri Lanka, a country with a population of approximately 23.3 million, currently has 12 medical faculties, all of which are affiliated with state universities and established by the government. Given that no private medical schools exist, undergraduate medical education is provided exclusively within the public sector. Four of these faculties were established after 2018 and remain in phases of active development and expansion. The Faculty of Medicine (FOM) at the University of Peradeniya (UOP), founded in 1962 and recognised as the second-oldest medical school in the country, has also experienced progressively increasing student intake [1]. The student population has grown despite the ongoing limitations in the nation’s physical infrastructure, technological capabilities, and academic personnel. These challenges have intensified due to the recent economic crisis faced by the country. Despite these constraints, Sri Lanka’s higher education sector continues to prioritise maintaining the quality of medical education and upholding national educational standards.

Traditional didactic lectures, particularly when delivered to large cohorts, are recognised as inadequate for cultivating clinical reasoning and the meaningful application of knowledge for some components of medical education [2, 3]. Collaborative learning can enhance critical thinking and critical reasoning skills in specific areas of medical education [4, 5]. This growing evidence reinforces the need to shift beyond purely lecture-based instruction toward more progressive, inclusive, and student-centred educational models that better reflect evolving learning environments and the diverse needs of contemporary medical students [6]. This is intensified by the fact that digital technologies, mobile devices, and generative artificial intelligence-driven tools make medical information widely available. This has changed what learners expect from education, making them want more interactive and application-based methods [7].

Team-based learning (TBL) originated in the 1980s through the work of Professor Larry Michaelsen in the United States, where it was initially developed for business education. It has become widely recognised in medical education as a pedagogically efficient, learner-centred method that supports active participation and effective use of resources [8]. TBL is a widely recognised active learning approach that fosters collaborative problem-solving, individual and group accountability, and the meaningful application of knowledge. An increasing corpus of academic research has demonstrated that TBL improves student engagement and fosters the cultivation of clinical reasoning abilities [9]. Nonetheless, most existing TBL models are tailored for small cohorts, require several facilitators, and presume the presence of considerable institutional resources. Consequently, the existing literature on the implementation of TBL in large cohorts is scarce, especially in LMICs contexts and in fields such as undergraduate obstetrics and gynaecology education [10]. Although some studies have explored the feasibility of TBL in large student groups [11, 12] there remains limited evidence on how TBL can be adapted for delivery within large lecture theatre settings using existing infrastructure and minimal faculty support. Evidence from both medical and other health professions’ education also supports the use of TBL as an effective approach to promote active learning and engagement across different disciplines [13].

Emerging digital interactive platforms such as “Pear Deck” could help address these constraints by allowing students to participate in real time, compare their work with that of their peers, and obtain feedback immediately in large classrooms [14]. Such technologies offer opportunities to adapt core TBL principles in a manner that maintains active learning while avoiding increases in faculty workload. The pilot intervention described in this study was developed following collaborative faculty development activities and experiential training in TBL conducted by the Department of Medical Education at the University of Illinois Chicago (UIC), United States of America (USA). This paper evaluates the feasibility and perceived value of a technology-supported TBL intervention delivered by a single lecturer to a large cohort of medical students within existing infrastructure, with contextual support from an international partnership. There is limited evidence on the feasibility of implementing team-based learning in large cohorts within resource-limited settings using minimal faculty support.

## Method

### Intervention development and study design

The Department of Medical Education, FOM, UOP, initiated this educational programme as part of a long-standing collaborative partnership with the Department of Medical Education, UIC, USA. For over six decades, UIC’s Department of Medical Education has played a pivotal role in the development and strengthening of the Department of Medical Education at the FOM UOP through ongoing academic support and joint initiatives [15]. As a recent milestone in this sustained collaboration, a three-day medical education training programme was conducted in March 2025 at the FOM, UOP, led by medical education specialists from the UIC, USA.

There were more than 40 participants in the training session. They included lecturers from the faculties of medicine and allied health sciences at Sri Lankan universities who were actively involved in teaching undergraduates. The programme introduced contemporary approaches in medical education, emphasising core TBL principles such as session design, team allocation, and the practical use of real-time interaction. During the training, TBL principles were demonstrated step by step, with participants divided into teams and a sample TBL session conducted. This supported capacity building in participants’ understanding and application of TBL principles. This collaborative training encouraged the authors to develop the pedagogical foundation required for the later introduction of TBL in the local educational setting using available resources and to implement this programme for medical undergraduates.

Following this training and further reading on guidance for TBL, faculty members independently design a pilot TBL intervention for a large group of medical undergraduates at the FOM, UOP [16, 17]. The objective was to convert the existing large-group didactic lecture into a TBL session. This study was conducted as a single-session pilot implementation and service evaluation at Kirkpatrick Level 1 (reaction), focusing on student satisfaction, engagement, and perceived relevance [18]. It employed descriptive methods to examine feasibility and student perceptions and was not designed or intended as an effectiveness or outcome trial.

### Educational context

The target group consisted of third-year medical undergraduates who had completed their pre- and para-clinical modules and were preparing to enter clinical rotations, where they would be expected to perform history taking, physical examinations, and clinical decision-making. Session preparation adhered to established TBL principles.

### Participants

A total of 252 students were systematically assigned to 20 teams. As all the students had successfully passed the barrier examination, their baseline knowledge of pre- and para-clinical subjects was considered comparable, eliminating the need for performance-stratified grouping. The team assignment was carried out digitally via Microsoft Excel to ensure randomisation. Teams were formed via a modulo-based allocation of student registration numbers. This process created twelve groups of 13 students and eight groups of 12 students, ensuring that the students were distributed as evenly as possible across the teams.

### TBL adaptation process

The Department of Obstetrics and Gynaecology facilitated the TBL session on abnormal uterine bleeding (AUB). AUB was chosen because it is a common clinical presentation that students are likely to encounter early in their clinical appointments. The learning objectives centred on developing structured clinical reasoning by integrating pre- and para-clinical knowledge, with an emphasis on symptom evaluation, the selection of appropriate investigations, and principles of patient management.

Eight simulated clinical case scenarios representing a comprehensive range of AUB, including adolescent, reproductive-age, postmenopausal, and iatrogenic presentations, were developed and given to students two weeks before the session. The students were informed that the activity would follow a team-based structure and that the session questions would be derived from these cases. The students were encouraged to review the relevant pre- and para-clinical material, with the expectation that this preparation would take two hours.

Additional learning materials, including recommended textbook chapters and relevant online resources, were provided. The students were also asked to bring internet-enabled smart devices so that they could take part in the live digital activities planned for the session. To further encourage readiness, a set of ten AUB-related multiple-choice questions (MCQs) was made available on the faculty learning management system (Moodle) as a self-assessment exercise. Immediate automated feedback with correct answers was provided. Lecturers spent approximately 10 h preparing the TBL session, including case development, creating MCQs for Moodle, and preparing materials. The case scenario and lecture presentation are provided in Supplementary File 1.

On the day of the session, the students were seated in the lecture theatre according to their assigned teams and asked to nominate a team leader. The structure, expectations, and flow of the TBL process were explained at the beginning of the study. The readiness assurance phase began with the Individual Readiness Assurance Test (iRAT). During this step, the first case scenario and its accompanying short-answer questions were shown to the students. Each scenario included between one and four questions, and students were given four minutes to complete their responses individually. The next was the Group Readiness Assurance Test (gRAT), in which teams were given five minutes to discuss their responses. Team leaders submitted their group responses via Pear Deck. Based on previous positive experiences from the authors of this paper, Pear Deck was selected to enhance real-time engagement by enabling live submission and display of student responses, thereby supporting simultaneous participation and discussion in large-group teaching [14]. The platform displayed the results in real time, along with the group identifiers. For many students, this was their first experience using Pear Deck, and it allowed them to compare their answers immediately, which encouraged discussion and engagement. Seeing how other teams responded prompted students to reflect more critically on their own reasoning. Following the gRAT for all participants, the facilitator guided the discussion for each case scenario. Microphones were used so that students across the large lecture theatre could contribute to the discussion.

The other seven case scenarios were discussed one after the other during the two-hour session. As the session progressed, teams appeared increasingly engaged, with some informal competitive dynamics observed (based on the facilitating lecturer’s qualitative impression), although these were not formally measured. With only one lecturer leading the session, they kept the large student group engaged by using digital tools and applying basic TBL principles. This structure enabled exploration of a wide spectrum of AUB presentations, including symptom appraisal, underlying physiological and pathological mechanisms, justification of investigations, and core management principles. Before the session ended, teams were given time to reflect on their peer evaluations, their individual roles, and how effectively they had worked together during the learning activities. A summary of this pilot TBL session is provided in Table 1. While the session incorporated key TBL components, a few adaptations were made to suit the local context. These included larger team sizes, delivery within a lecture theatre setting, and the use of a digital platform for response submission.

**Table 1:** Implementation of TBL components in the pilot session

TBL principle	Implementation in the present session
Preparatory phase	• Eight AUB case scenarios distributed two weeks prior to the session • Recommended textbook chapters and online resources provided • Ten MCQs administered via Moodle as self-assessment with automated feedback • Students advised to spend 1–2 h preparing and to bring internet-enabled devices
Individual Readiness Assurance Test (iRAT)	• Short-answer questions (3–5 per case) displayed for individual response • Four minutes allocated for independent completion • Focus on recall and application of pre- and para-clinical knowledge
Group Readiness Assurance Test (gRAT)	• Five minutes allocated for structured team discussion • Team leaders submitted responses via Pear Deck • Real-time display of group answers with identifiers • Immediate comparison of responses across teams
Whole-batch discussion and feedback	• Facilitator-led clarification after each case • Use of microphones to enable contribution in a large lecture theatre • Reflection on reasoning by comparing peer responses • Sequential discussion of eight cases within two-hour session
Peer feedback and reflection within teams	• Teams reflected on preparation and individual contributions at session end • Discussion of collaborative learning process • Encouragement of self-awareness regarding knowledge gaps

*MCQ *Multiple choice questions

### Instruments and outcome measures

The students’ views on the TBL session were collected using a structured online questionnaire distributed through Google Forms. The instrument comprises ten items on a Likert scale, focused on the recently conducted TBL session, eliciting recommendations for preparation and improvements for future sessions. Each items rated on a scale from 1 (strongly disagree) to 5 (strongly agree). Optional free-text responses provided additional qualitative insights. The evaluation was conducted at Kirkpatrick Level 1, focusing on students’ reactions and perceptions of the TBL session. The questionnaire was developed for this pilot study, with expert consensus among faculty members with experience in medical education to ensure content relevance and formal pilot testing or psychometric validation was not performed. The questionnaire items were designed to capture several distinct aspects of students’ perceptions and suggestions, such as engagement, teamwork, preparation, views on the digital platform, and ideas for future planning, rather than to form a single underlying construct. Therefore, internal consistency measures such as Cronbach’s alpha were not considered appropriate. The questionnaire was available only in English, and the Likert-scale items are presented in Table 2. The full version of the questionnaire is provided in Supplementary File 2.

**Table 2:** Questionnaire items used to assess student perceptions of the TBL session

Item number	Statement
1	The TBL session kept me actively involved.
2	I prepared actively for the TBL session prior to attending.
3	Pre-session self-assessment improved my team participation.
4	Group discussions helped me learn from my peers’ perspectives.
5	The platform (Pear Deck) improved interaction in a large class.
6	I had sufficient chance to contribute to my team.
7	I participated more than in a traditional lecture.
8	This method is suitable for large groups.
9	This approach encouraged deeper learning.
10	I would like more teaching sessions to be conducted using this TBL.

### Analysis

As this was a pilot study, data were analysed using descriptive statistics only, including medians and interquartile ranges. Inferential statistical analyses were not conducted because the study was designed to provide descriptive insights into feasibility and student perceptions, without comparison groups or hypothesis testing. These values were calculated using IBM SPSS Statistics, version 31 (v31). Free-text responses were analysed using a simple thematic approach. Responses were reviewed, coded, and grouped into recurring themes by the first author, with review by a second author to ensure consistency.

### Ethical considerations

This study was conducted as a service evaluation in accordance with institutional guidelines and did not require formal Research Ethics Committee approval. Approval was obtained from the Department of Medical Education, FOM, UOP. Further details are provided in the “Ethics approval and consent to participate” section.

## Results

A total of 252 third-year medical students were enrolled and eligible for the TBL session, and 209 attended (82.9%). This participation rate is comparable to typical lecture attendance. At our institution, attendance is monitored at the individual level across the entire course, with a minimum overall requirement of 80%, rather than being mandated for specific sessions. Pear Deck enabled participation at the team level, with each team submitting responses through a designated team member, and showed that all 20 teams remained engaged and connected throughout the session. Before submitting their answers, each team took time to talk through the questions together. During the facilitated discussion, each team was allowed to share and justify their reasoning aloud. All of the planned case scenarios were discussed and completed within the scheduled two-hour session.

**Fig. 1:**
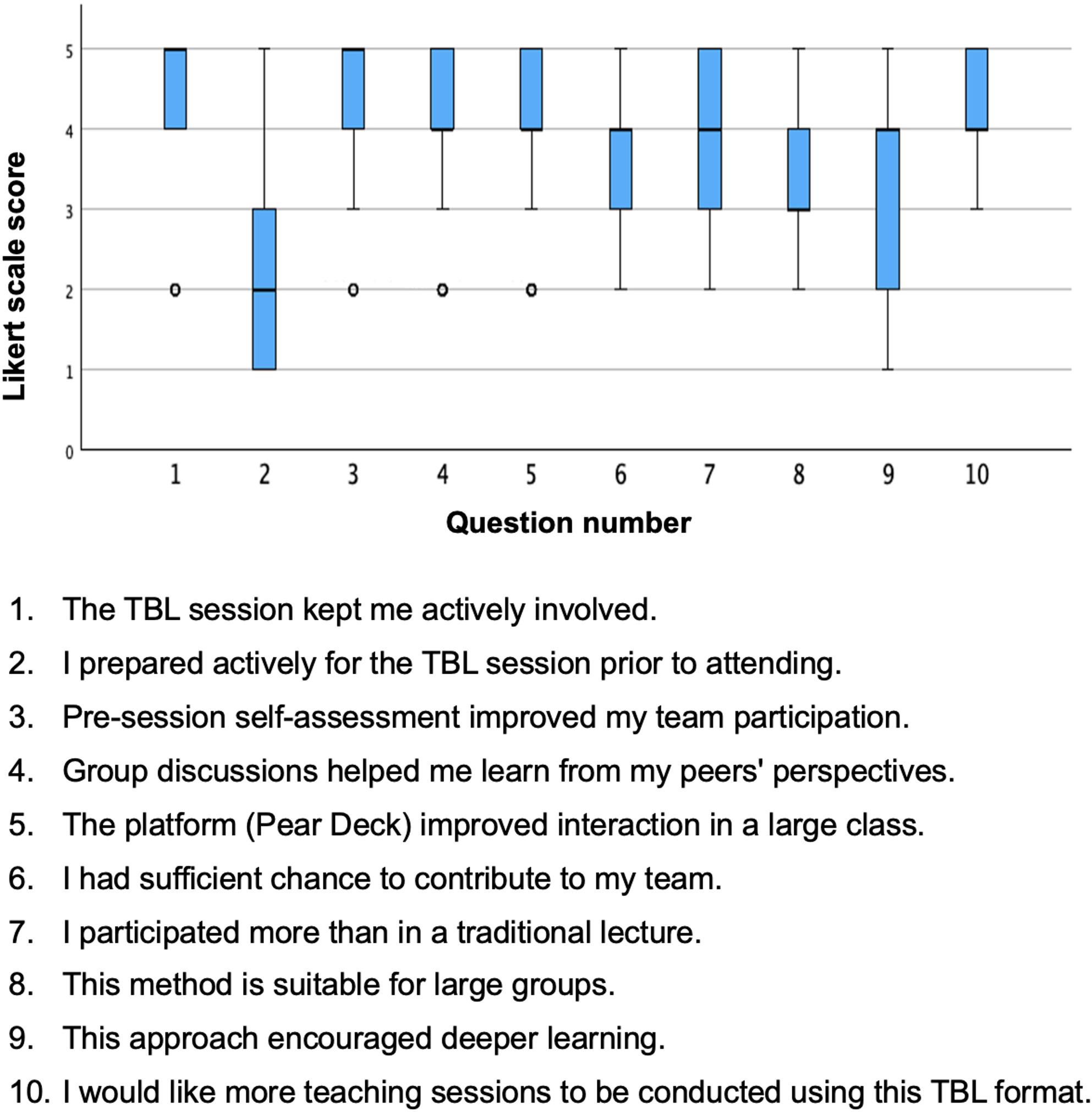
Distribution of student responses to the TBL session questionnaire. Responses to ten Likert-scale items (1= strongly disagree, 2=disagree, 3=neutral, 4= agree, 5= strongly agree) are presented as box-and-whisker plots. The central line within each box represents the median, the box indicates the interquartile range, whiskers represent the range, and circles denote outliers

Among 209 students who took part in the session, 191 completed the survey, resulting in a response rate of 91.4%. Responses from non-attending students were not considered for the analysis. The distribution of responses for each questionnaire item is presented in Fig. 1.

Analysis of the Likert-scale responses revealed predominantly positive perceptions across the questionnaire items. Most items had median ratings of 4 or higher, indicating that many students expressed agreement with statements related to their engagement, teamwork, and the perceived value of the digital platform for learning. The students also described feeling actively involved, noted greater participation than traditional lectures did, and reported benefits from both the group discussions and the preparatory activities. Conversely, the ratings for pre-session preparation were lower, indicating that students engaged with this aspect of the TBL approach in various ways. Opinions varied regarding the usefulness of TBL for large groups, with many individuals opting for neutral responses. Taken together, these trends highlight the elements of the session that received strong student endorsement while also indicating those areas in which student perceptions were more heterogeneous.

A total of 52 students were provided free comments, and they were analysed using a simple descriptive thematic approach. Analysis of the responses identified three main themes: engagement and interactivity, teamwork and collaboration, and reflection on learning. As this was a pilot evaluation, achieving qualitative saturation was not an objective. Students often characterised the activity as both engaging and interactive, and several commented that it helped them sustain their attention throughout. The comments also reflected favourable attitudes towards team-based discussions, which promoted self-reflection and recognition of knowledge gaps. Some of the students’ free responses are as follows:

“The session was very interactive. Because of that, I could stay focused from beginning to end.”

“Usually, after lunch lectures I feel sleepy, but this time I did not feel sleepy.”

“Teamwork was good and fun. I realised that my knowledge is not enough in some areas.”

## Discussion

This study reports on the feasibility of implementing a technology-enhanced TBL session delivered for a large cohort of medical undergraduates in a resource-limited setting. The findings indicate that the adapted TBL approach was feasible, acceptable to students, and deliverable within existing institutional structures. Students reported high levels of engagement and positive perceptions of the session. It was reported that the digital platform played a central role in keeping students actively involved, enabling teams to work collaboratively in real time despite the constraints of a large lecture theatre. However, perceptions of TBL’s suitability for large groups were more varied, with neutral median ratings, indicating that although the approach was feasible, students’ experiences differed. In addition, the lower scores for pre-session preparation suggest that students engaged with this part of the TBL process in different ways. This highlights the importance of building in clearer supports or incentives to help ensure more consistent preparation in future sessions. This study examines the feasibility of implementing the TBL intervention for a large cohort of medical undergraduates using existing instructors, while building staff capacity in TBL through international academic training, rather than assessing the broader impact of the international partnership. Overall, this approach appears feasible and is perceived by students as supportive of active learning within this specific educational context.

A key takeaway from this intervention is that a single lecturer can deliver an organised TBL session to a large cohort. From the facilitator’s perspective, the session was feasible for a single lecturer in a lecture theatre, although preparation was time-intensive; however, only a small number of lecturers were involved in this pilot. To our knowledge, there is limited published evidence on implementing TBL in large cohorts or modifying the standard TBL steps [11, 12], highlighting a gap in the literature. In contrast to previous works, this study specifically examines how TBL can be adapted for large-group delivery within the constraints of an existing lecture theatre. In Sri Lanka, where high student-to-staff ratios and limited resources are common, adapting existing teaching approaches is essential to maintaining active learning. The digital tools used in this session enabled simultaneous contributions from all teams and supported a smooth workflow, demonstrating how technology can make interactive learning feasible without additional facilitators. Experiences from other LMIC settings echo this finding, demonstrating that TBL can still succeed even when staffing and infrastructure are stretched and suggesting that TBL approaches may be a feasible form of active learning under such constraints [19]. However, further studies are needed to assess long-term sustainability, cost implications, and scalability in similar contexts. These findings should be interpreted within the context of this single-session pilot study and primarily reflect feasibility and student perceptions rather than broader educational outcomes.

The collaborative faculty development initiative with a partner institution in a high-income country played an important role in shaping this intervention. Exposure to current medical education practices, combined with structured mentorship, helped the local team adapt TBL principles in ways that suited the resources and constraints of the local context. Such partnerships can support educational innovation in LMICs by promoting knowledge sharing and facilitating the implementation of new ideas in specific local settings. The literature provides examples of how international partnerships have contributed to the development of various disciplines in medical education [20–22].

This study has several methodological limitations that should be taken into account. It was conducted as a single-session pilot on a single topic, without a comparison group, and no pre-post outcome measures were collected. The evaluation also focused mainly on feasibility and student perceptions of TBL rather than on objective indicators of learning or clinical performance. As a result, the findings should be viewed as exploratory, reflecting student engagement and acceptability rather than demonstrating measurable gains in knowledge or clinical reasoning. Students’ perceptions may have been influenced by the specific topic and its level of difficulty. In addition, the questionnaire was not designed as a single-scale instrument, and formal validation, including internal consistency testing, was not performed. This lack of validation should be considered when interpreting the findings. Although the digital platform supported interaction, its specific contribution to learning was not formally evaluated. Student attendance for the study was 82.9%. However, the reasons for non-attendance were not explored, and the possibility of participation bias cannot be excluded. Furthermore, the survey responses reflect the views of only the 91.38% of students who attended the session and may not represent the entire cohort.

Also, limitations were noted in relation to established TBL standards. The size of the overall cohort resulted in teams that were larger than those typically described in the literature, which may have implications for individual accountability and opportunities for participation [23]. Larger teams can also increase the likelihood of unequal participation, with some students being less engaged. In addition, the fixed seating configuration of the lecture hall restricted face-to-face interaction within teams, potentially affecting the quality and depth of group discussions [24]. Resource constraints also made it unfeasible to implement certain standard elements of the TBL, such as scratch-off cards for readiness assurance testing. As a result, it was not possible to use every aspect of the standard TBL process to its full extent. Given these constraints, particularly as a single-centre pilot conducted in a resource-limited context, the findings may not be generalisable to other settings.

### Future implications for practice

Future work should aim to refine and extend this approach. Although a single lecturer facilitated the session, preparation was time-intensive, requiring approximately 10 h. The sustainability of this approach may depend on workload distribution and faculty availability in routine practice. Holding TBL sessions in smaller tutorial spaces and involving additional facilitators may help improve interaction and align delivery more closely with recognised TBL standards. Ongoing investment in faculty development will also be important to ensure that staff are equipped to design TBL materials, guide team activities, and use digital tools effectively. More intentional incorporation of TBL principles into the curriculum may enhance students’ preparation and engagement, with further refinement supported by collaboration with the partner institution and guidance available when required through an established, longstanding academic relationship. This study examined students’ perceptions of engagement and acceptability; future evaluations that include cognitive load, clarity of expectations, and perceived fairness may provide a more comprehensive understanding of the learning experience. Continuous professional development, coupled with supportive institutional policies, has the potential to encourage more staff to implement active learning strategies in a resource-constrained environment. Furthermore, this approach may contribute to the sustainability and expansion of the model over time.

## Supplementary Information

Supplementary Material 1.

Supplementary Material 2.

## Data Availability

Data supporting the findings of this study are available from the corresponding author on reasonable request.

## Abbreviations

LMICs: Low- and middle-income countries
TBL: Team-Based Learning
FOM: Faculty of Medicine
UOP: University of Peradeniya
UCI: University of Illinois Chicago
USA: United States of America
AUB: Abnormal Uterine Bleeding
iRAT: Individual Readiness Assurance Test
gRAT: group Readiness Assurance Test
MCQ: Multiple-Choice Question
