# Supplementary material for: Implementing digital-supported team-based learning for large undergraduate cohort in a resource-limited setting: a pilot study developed through an international academic partnership
Source: BMC Med Educ. 2026 May 9;26:1053. doi: 10.1186/s12909-026-09409-y (PMC13326472; doi:10.1186/s12909-026-09409-y)
Supplement: Supplementary file 1 — Supplementary Material 1. [file 12909_2026_9409_MOESM1_ESM.pdf]

Abnormal Uterine Bleeding
IAM II
Team Based Learning (TBL)

Dr Neranja Fonseka

Lecturer
Department of Obstetrics and Gynecology
Faculty of Medicine
University of Peradeniya
Consultant in Obstetrics and Gynecology (Acting)
Teaching Hospital Peradeniya

Case no 1

Ms. Malika is a 40-year-old mother of two children, both delivered vaginally without complications. She works long shifts at a garment factory to support her family. Until the age of 39, her menstrual cycles were regular (every 28–30 days, lasting 5 days). However, over the past year, her periods have become **excessively heavy,** requiring double sanitary pads and frequent changes (every 1–2 hours). She passes **large clots,** often staining her clothes, which embarrasses her at work.

Ms. Malika is a 40-year-old mother of two children, both delivered vaginally without complications. She works long shifts at a garment factory to support her family. Until the age of 39, her menstrual cycles were regular (every 28–30 days, lasting 5 days). However, over the past year, her periods have become **excessively heavy,** requiring double sanitary pads and frequent changes (every 1–2 hours). She passes **large clots,** often staining her clothes, which embarrasses her at work.

Case no 1

She feels **constantly fatigued**, dizzy, and has developed unusual cravings for raw rice. Mentally, she feels **anxious and depressed,** frustrated by her inability to function normally. A cervical smear at 35 was normal, and she has no personal or family history of bleeding disorders or cancers. She has no fertility wishes and is not on contraception.

Case no 1

1. Define Abnormal Uterine Bleeding (AUB)
2. How this affects her quality of life.
3. Explain the pathophysiology of the presence of clots in menstrual blood.
4. What complications has she developed?

Students, write your response!

Pear Deck Interactive Slide
Do not remove this bar

AUB

Abnormal uterine bleeding is defined as bleeding from the uterine corpus that is abnormal in regularity, volume, frequency, or duration and occurs in the absence of pregnancy.

-FIGO 2011-

AUB

May be

- Chronic - hasbeen present for the majority of the **past 6 months**.
- Acute -defined as an episode of heavy bleeding that, in the opinion of the clinician, is of sufficient quantity to require immediate intervention to prevent further blood loss

- FOGO 2011-

Case no 2

Ms. Rina, a 35-year-old unmarried schoolteacher, presents with **prolonged menstrual bleeding** over the past year while maintaining a regular 28-day cycle. She requires hourly pad changes on heavy days, passing **large clots,** but reports **no significant pain**. Recently, she has also noticed **frequent urination** (no diabetes).

On examination, a **pelvic mass** is palpable, compatible with a **20-week-size uterus,** with an **irregular surface**. Despite being medically fit and not depressed, her main concern is her **upcoming marriage**. She worries about how her condition will affect marital life, intimacy, and future fertility.

Case no 2

What is the most likely cause of AUB clinically?

Case no 2

What is the most likely cause of AUB clinically?

Clues

- prolonged menstrual bleeding over the past year while maintaining regular cycles
- Presence of pressure symptoms
- Large pelvic mass with irregular surface
- Nulliparity

Students, write your response!

Pear Deck Interactive Slide
Do not remove this bar

Leiomyoma

Benign fibromuscular tumors of the myometrium

Lesions are dependent on the presence of the sex steroids, **oestrogen** and **progesterone**

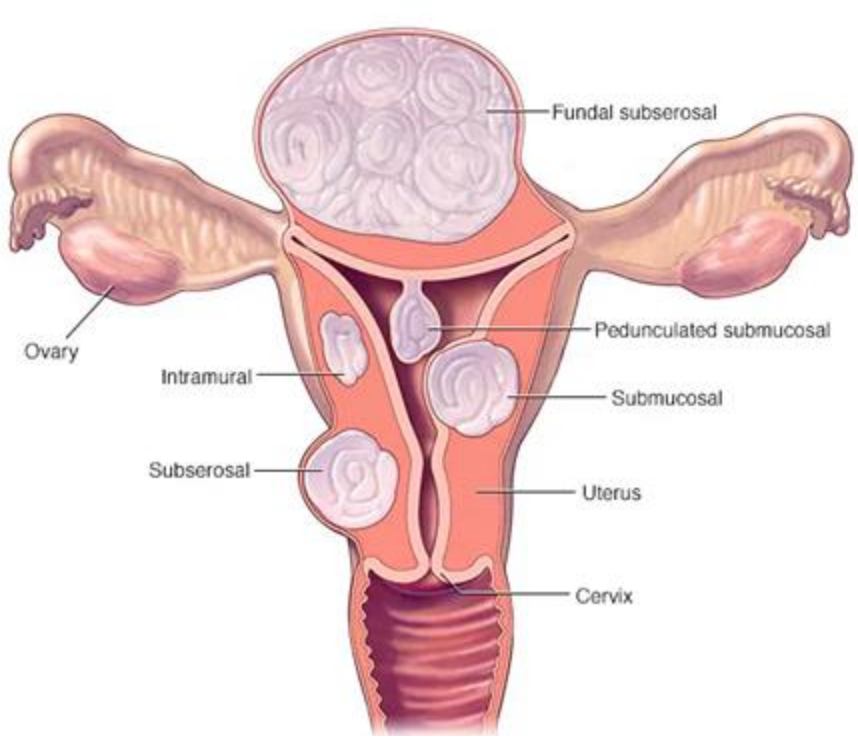

Case no 2

1. What investigations would you like to offer?

2. What treatment options would you like to discuss with her?

Students, write your response!

Pear Deck Interactive Slide
Do not remove this bar

Case no 2

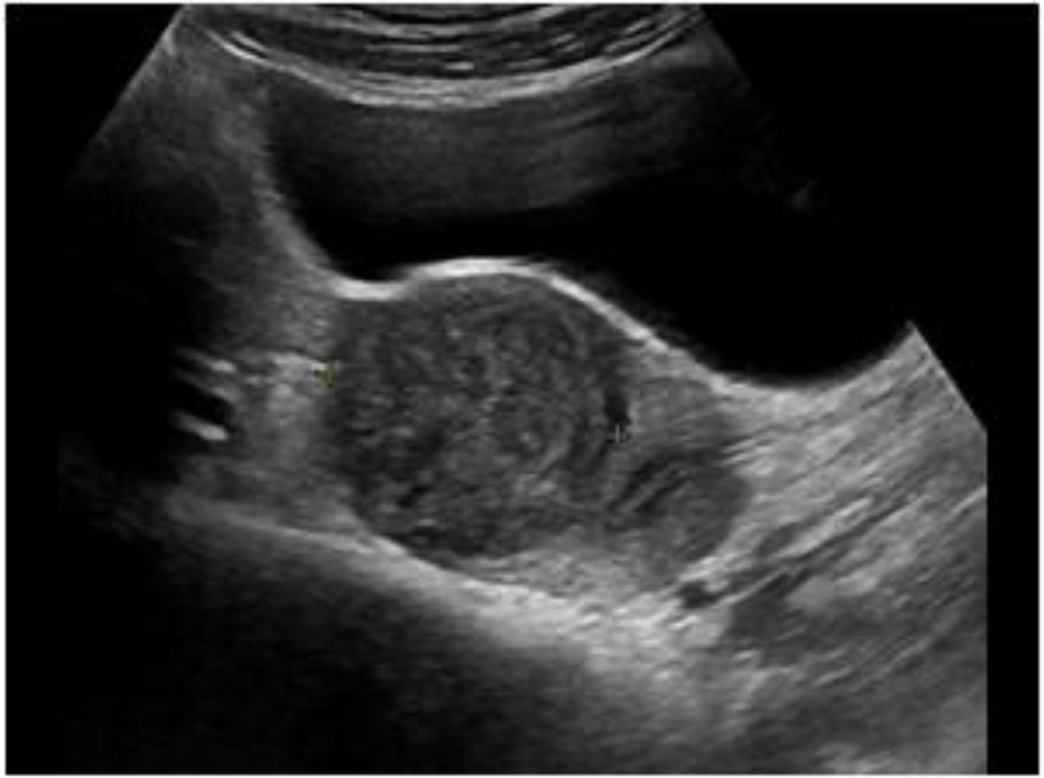

Case no 3

Ms. Kumari, a 44-year-old housewife and **mother of three**, reports progressively worsening menstrual bleeding over two years, now soaking 6-8 pads/day with large clots and prolonged 8-10 day episodes. She experiences **severe cramping pelvic pain radiating to her back and thighs, starting pre-menses and persisting post-bleeding**. The symptoms force her to change pads hourly, frequently stain clothes, and prevent household chores and social activities. She avoids intimacy due to pain and bleeding concerns. Examination reveals a u**niformly enlarged, mildly tender uterus (10-12 weeks size)** with normal cervix. Though using condoms occasionally with **no fertility desires**, she's frustrated by how symptoms disrupt her family care and social life, seeking effective treatment.

Case no 3

What is the most likely cause of AUB clinically?

Students, write your response!

Pear Deck Interactive Slide
Do not remove this bar

Case no 3

What is the most likely cause of AUB clinically?

Clues

- HMB
- IIry dysmenorrhea
- Age 44 yrs
- Multiparity
- Uniformly enlarged uterus

Adenomyosis

presence of endometrial tissue, including endometrial **glands** and **stroma**, in the myometrium

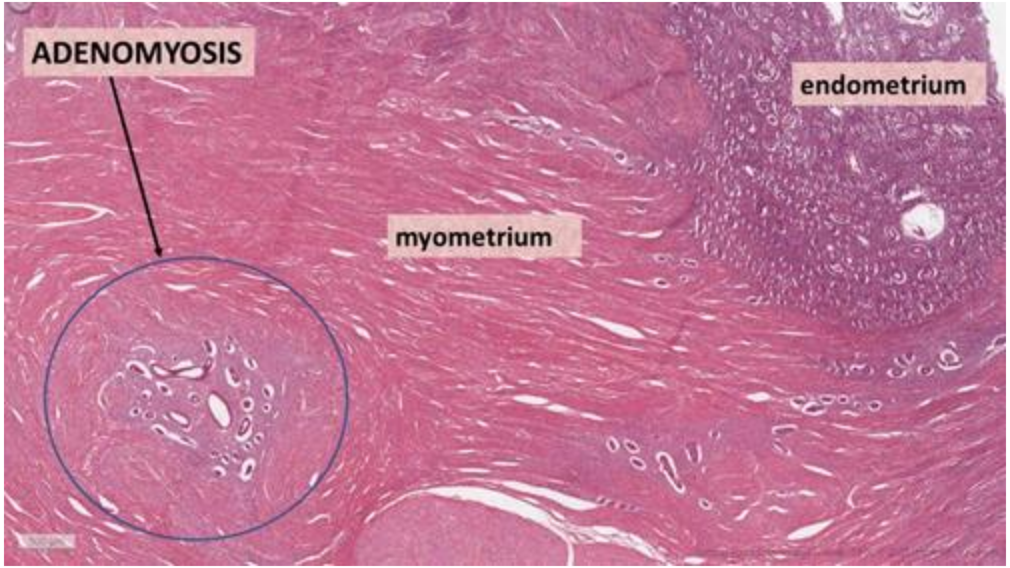

Case no 3

1. What investigations would you like to offer?

2. What treatment options would you like to discuss with her?

Students, enter a number!

Pear Deck Interactive Slide
Do not remove this bar

Case no 4

Ann, a 30-year-old married woman with **primary subfertility**, presents with **irregular menstrual cycles occurring only once every 3 months**. When menstruation does occur, she experiences excessive bleeding requiring pad changes every 2 hours with frequent passage of clots, though she reports no significant pain. She was diagnosed with **PCOS** during fertility investigations. On examination, Ann is obese with a **BMI of 35.6kg/m^2** and shows signs of hirsutism, acanthosis nigricans and acne. **A pelvic ultrasound performed before menstruation revealed a thickened endometrium** and polycystic ovaries. Her primary concerns are the unpredictable nature of her menstrual cycles and her ongoing difficulties conceiving, which are causing significant distress in her personal life.

Case no 4

Explain the pathophysiological basis for the following symptoms and investigation findings in this patient.

1. irregular menstrual cycles occurring only once every 3 months
2. when menstruation does occur, she experiences excessive bleeding
3. thickened endometrium

Students, write your response!

Pear Deck Interactive Slide
Do not remove this bar

Case no 5

Mrs Damayanthi, a 56-year-old woman, presented with her first episode of **postmenopausal bleeding** after 6 years of amenorrhea. She has a history of **poorly controlled diabetes and hypertension**, with o**besity (BMI 34.8)**. Previously, she had regular menstrual cycles without significant gynaecological issues and is a mother of three children, all delivered vaginally. Her last cervical smear, performed two years ago, was normal, and there is no family history of breast, ovarian, colonic, or endometrial cancer.

On examination, her cervix appeared normal. Transvaginal ultrasound revealed a t**hickened endometrium measuring 8 mm.** A Pipelle endometrial biopsy was performed, which showed **endometrial hyperplasia with atypia**. Mrs. Damayanthi is concerned about the unexpected bleeding and the biopsy results, particularly given her underlying metabolic conditions.

Case no 5

1. Define endometrial hyperplasia.
2. Describe the classification system used for endometrial hyperplasia.
3. What are the risk factors for the above condition?
4. What is the best treatment option?

Students, write your response!

Pear Deck Interactive Slide
Do not remove this bar

Case no 5

proliferation of the endometrial glands with an increase in the gland to stroma ratio when compared to proliferative endometrium

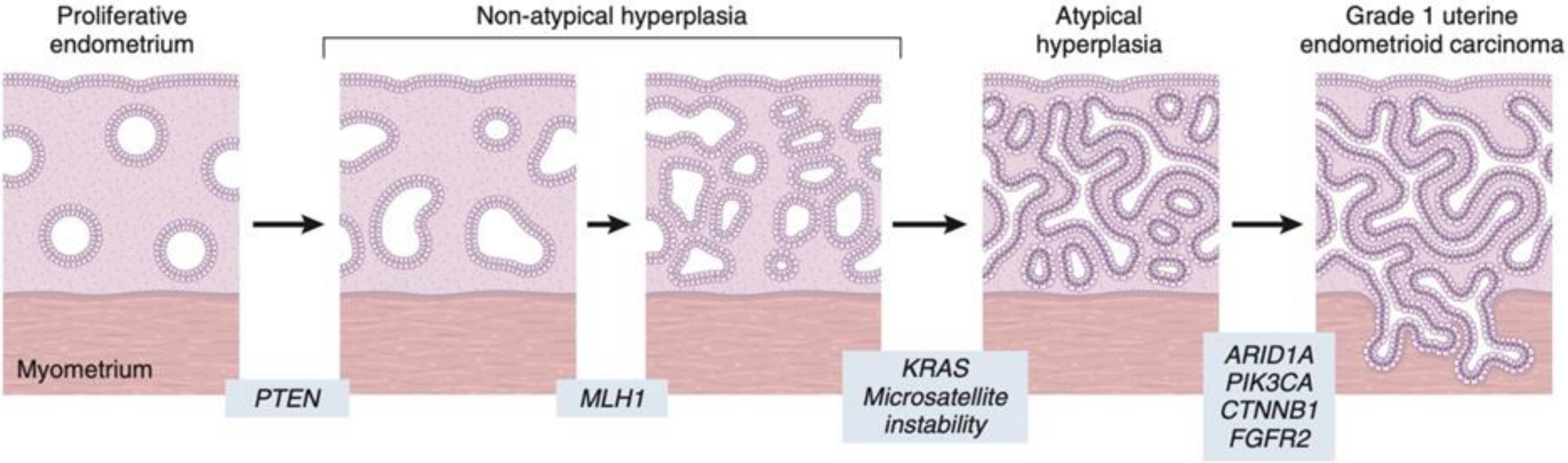

Case no 5

WHO classification- two types

I. Endometrial hyperplasia without atypia
II. Endometrial hyperplasia with atypia/ Atypical hyperplasia

Endometrial hyperplasia develops when estrogen, unopposed by progesterone

Case no 6

Riya, a 15-year-old student, experienced menarche at age 12 and has **since** struggled with prolonged, heavy bleeding lasting up to 10 days, accompanied by large clots. The excessive bleeding forces her to miss school frequently, significantly impacting her studies. Further history reveals **associated bleeding gums during toothbrushing and easy bruising with minor trauma**. There is no family history of bleeding disorders.

Otherwise, she is medically fit. A pelvic ultrasound shows a normal-sized uterus. Laboratory tests reveal mild anaemia (Hb 9.8 g/dL). Riya is distressed by the disruption to her education and social life due to unpredictable, debilitating bleeding episodes.

Case no 6

1. What are the possible causes of AUB?

2. What additional investigations would you like to offer?

Students, write your response!

Pear Deck Interactive Slide
Do not remove this bar

Case no 6

1. Von Willebrand disease (VWD)

   Immune thrombocytopenia (ITP)

   Inherited factor VIII and factor IX coagulation defects

   Factor II, V, VII, X and XIII deficiencies- rare

1. Blood picture

   coagulation screen including fibrinogen, PT and aPTT

   Factor assay

Case no 7

Kumari, a 39-year-old mother of three, is a housewife. She has no medical problems and maintains regular 28–30-day menstrual cycles. For the past month, she has experienced **painless bleeding after intercourse**, which is affecting her sexual life. Her last cervical **smear at age 35 was normal.** She underwent tubal ligation during her third cesarean delivery. She is concerned about this new symptom and its implications, though she reports no other gynaecological issues

Case no 7

1. Where would the pathology be?

2. What examination would you like to do?

Students, write your response!

Pear Deck Interactive Slide
Do not remove this bar

Case no 7

Postcoital bleeding typically arises from contact with lesions on the cervix, vagina or vulva

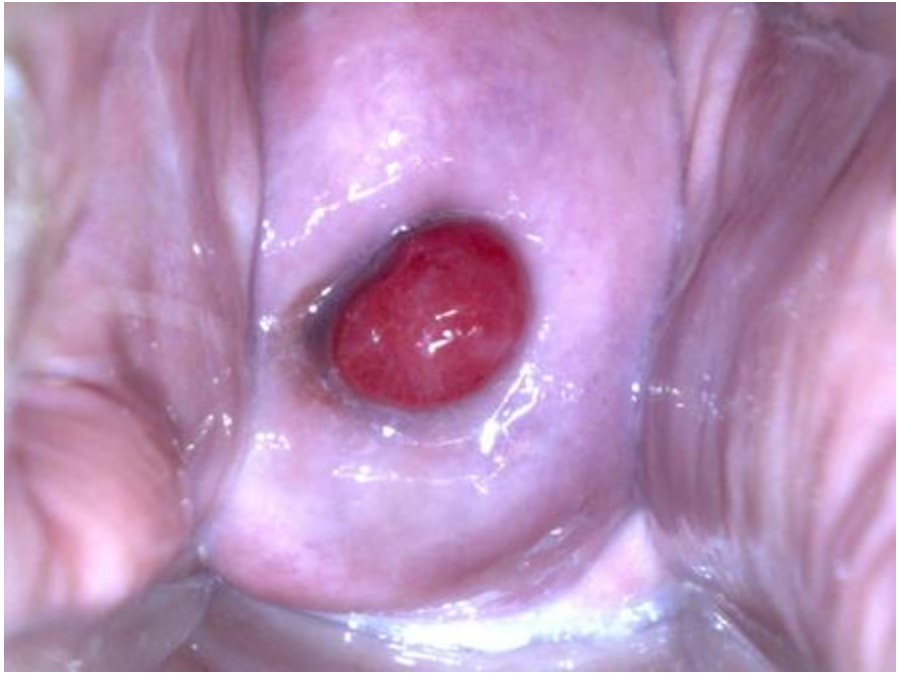

Case no 7

The lesions are usually benign but a small minority may have atypical or malignant features

How would you manage?

Case no 8

Priya, a healthy 46-year-old mother of two adult children (18 and 21 years), has been using **DMPA** (Depot medroxyprogesterone acetate) for contraception for 3 years. While initially **experiencing amenorrhea for 6 months**, she now complains of unpredictable, light but disruptive bleeding episodes that interfere with her daily activities. She has no medical issues (BMI 26) or a family history of cancers. Her last cervical smear at 45 years old was normal, and she reports no vasomotor symptoms. Ultrasound shows a n**ormal-sized uterus with thin endometrium**, and her haemoglobin is 11.1 g/dL. Priya is concerned about this new bleeding pattern while wanting to continue contraception.

Case no 8

1. Explain the pathophysiology of the changes in menstruation.

2. Explain how you would manage.

Students, write your response!

Pear Deck Interactive Slide
Do not remove this bar
