# Supplementary material for: Implementing digital-supported team-based learning for large undergraduate cohort in a resource-limited setting: a pilot study developed through an international academic partnership
Source: BMC Med Educ. 2026 May 9;26:1053. doi: 10.1186/s12909-026-09409-y (PMC13326472; doi:10.1186/s12909-026-09409-y)
Supplement: Supplementary file 2 — Supplementary Material 2. [file 12909_2026_9409_MOESM2_ESM.pdf]

Student Evaluation of the Team-Based Learning Session

Completion of this questionnaire is voluntary and anonymous.

1. Please select the response that best reflects your opinion for each statement below.

Mark only one oval per row.

	1. Strongly disagree	2. Disagree	3. Neutral	4. Agree	5. Strongly agree
**The TBL session kept me actively involved.**	⬭	⬭	⬭	⬭	⬭
**I prepared actively for the TBL session prior to attending.**	⬭	⬭	⬭	⬭	⬭
**Pre-session self-assessment improved my team participation.**	⬭	⬭	⬭	⬭	⬭
**Group discussions helped me learn from my peers' perspectives.**	⬭	⬭	⬭	⬭	⬭
**The platform (Pear Deck) improved interaction in a large class.**	⬭	⬭	⬭	⬭	⬭
**I had sufficient chance to contribute to my team.**	⬭	⬭	⬭	⬭	⬭
**I participated more than in a traditional lecture.**	⬭	⬭	⬭	⬭	⬭
**This method is suitable for large groups.**	⬭	⬭	⬭	⬭	⬭
**This approach encouraged deeper learning.**	⬭	⬭	⬭	⬭	⬭
**I would like more teaching sessions to be conducted using this TBL format.**	⬭	⬭	⬭	⬭	⬭

2. Any additional comments or suggestions?

____
